# Effects of urban motorways on physical activity and sedentary behaviour in local residents: a natural experimental study

**DOI:** 10.1186/s12966-017-0557-0

**Published:** 2017-07-27

**Authors:** R. G. Prins, L. Foley, N. Mutrie, D. B. Ogilvie, on behalf of the M74 study team

**Affiliations:** 1 MRC Epidemiology Unit and UKCRC Centre for Diet and Activity Research, University of Cambridge, School of Clinical Medicine, Box 285 Institute of Metabolic Science Cambridge Biomedical Campus, Cambridge, CB2 0QQ UK; 2 Physical Activity for Health Research Centre, Institute of Sport, Physical Education and Health Sciences, University of Edinburgh, Edinburgh, UK

**Keywords:** Physical activity, Natural experimental study, Health behaviour, Environment, Urban planning, Urban renewal

## Abstract

**Background:**

There is little evidence on how changing the physical environment changes health-related behaviours. We studied the effects of the new M74 motorway (freeway) — opened in 2011 — and the existing M8 motorway in Glasgow, Scotland, on physical activity and sedentary behaviour among local residents.

**Methods:**

This natural experimental study used baseline (T1; 2005) and follow-up data (T2; 2013) from a longitudinal cohort (*N* = 365) and two cross-sectional samples (T1 *N* = 980; T2 *N* = 978). Adult participants were recruited from three study areas: one surrounding the new motorway, one surrounding the existing motorway, and a third, control, area without a motorway. The outcomes were self-reported time spent sitting, walking, and in moderate-to-vigorous physical activity (MVPA). Motorway exposure was defined in terms of (1) study area and (2) distance from home to the nearest motorway junction. Outcomes were regressed on exposures in two-part (walking and MVPA) or linear (sedentary behaviour) cohort and repeat cross-sectional models, adjusted for baseline behaviour and sociodemographic covariates.

**Results:**

Cohort participants living in the M8 area were less likely to participate in MVPA at follow-up than those living in the area without a motorway (OR 0.37; 95%CI 0.15, 0.91). Within the M8 area, those living closer to the motorway were also less likely to do so (OR 0.30; 95%CI 0.09, 0.97). No other statistically significant results were found.

**Conclusions:**

We found some evidence of a negative association between exposure to an existing urban motorway and MVPA. However, the behavioural impacts of motorways are likely to be complex and evolve over time.

**Electronic supplementary material:**

The online version of this article (doi:10.1186/s12966-017-0557-0) contains supplementary material, which is available to authorized users.

## Background

Physical activity is important for health and wellbeing and may help prevent a wide range of non-communicable diseases [1]. Sedentary behaviour has been associated, independently of physical activity, with both cardiovascular [2] and all-cause [3, 4] mortality. Globally, however, a third of adults are estimated to be physically ‘inactive’ (i.e. not meeting any of three criteria: 30 min of moderate-to-vigorous physical activity (MVPA) on at least five days per week, 20 min of vigorous physical activity on at least three days per week, or not achieving 600 MET-minutes per week) and four in ten sit for more than four hours per day [5]. Physical inactivity and sedentary behaviour are particularly prevalent in more affluent countries. In Scotland, for example, adults are sedentary for 5.5 h per day on average and four in ten are ‘insufficiently’ active [6]. In the latter study adults were defined as ‘sufficiently’ active if they engaged in MVPA for a minimum of 150 min per week or engaged in 75 min of vigorous activity per week, and carried out activities that strengthen muscles on at least two days per week.

Physical activity and sedentary behaviour are partly shaped by local physical environmental conditions, such as the availability of recreational facilities and infrastructural design [7, 8]. Changes in the physical environment, for example to road infrastructure, may influence physical activity and sedentary behaviour in local communities. For example, providing more or better roads may encourage car use, which may increase the time spent in sedentary travel. In addition, more traffic in local streets may make it less safe and attractive for people to be physically active outdoors, and environments that are less conducive to physical activity may promote increases in time spent sedentary [9]. On the other hand, improving (road) access to more distant recreational amenities may facilitate their use [10]. Overall, the evidence either way for the effects of new road infrastructure on physical activity is scant [11] and there are no studies of its impact on sedentary behaviour.

One reason for this is that it can be difficult to evaluate interventions for which the intervention design and implementation are out of the researcher’s control and randomisation is either not feasible or not ethical. Such interventions can be seen as natural experiments, which are not designed for research purposes but provide valuable opportunities to understand the impact of environmental change on health behaviour [12]. One such opportunity was the construction of an eight-kilometre extension to the M74 motorway (freeway) in Glasgow, Scotland. This new, six-lane section of urban motorway runs through some of the most deprived neighbourhoods in Glasgow, Scotland and Europe. The new motorway was intended to relieve through traffic on an existing urban motorway, the M8. However, this infrastructural project was also intended to promote economic regeneration, and was hypothesized to remove traffic from local streets. Proponents of the scheme argued that the motorway would create a more pedestrian- and cycle-friendly environment. In contrast, detractors argued that the motorway would increase local traffic and encourage use of motor vehicles in the affected neighbourhoods, discouraging local walking and cycling trips. These narratives have previously been summarised as two contrasting overarching hypotheses [13] which formed the basis for a natural experimental study of the effects of exposure to urban motorway infrastructure on a range of public health outcomes. In this paper, we focus on changes in physical activity and sedentary behaviour over time, thereby aiming to address the comparative lack of evidence of the effects of major road infrastructure on these health behaviours.

## Methods

### Intervention, study design and sample

#### Intervention

A detailed description of the intervention has been published elsewhere [13]. Briefly, since the 1960s the M8 motorway has passed through Glasgow, Scotland’s largest city. Another motorway, the M74, ended at the southeastern edge of the city. In 2008, construction began to build an eight-kilometre (five-mile), six-lane extension of the M74 motorway to make an additional connection to the M8. The M74 extension opened in June 2011.

#### Study design

The effects of the new motorway on patterns of travel, physical activity and wellbeing in local residents were evaluated using a quasi-experimental study design. The study entailed a longitudinal cohort and two cross-sectional samples (baseline (2005) and follow-up (2013)). In the longitudinal cohort *intra-individual change* over time was studied, which provided the greatest support for causal inference. In the repeated cross-sectional samples *population shifts over time* in the outcomes were examined. This increased the sample size and insight into population-level changes not captured in the cohort, but with less confidence for causal inference at an individual level.

**Fig. 1:**
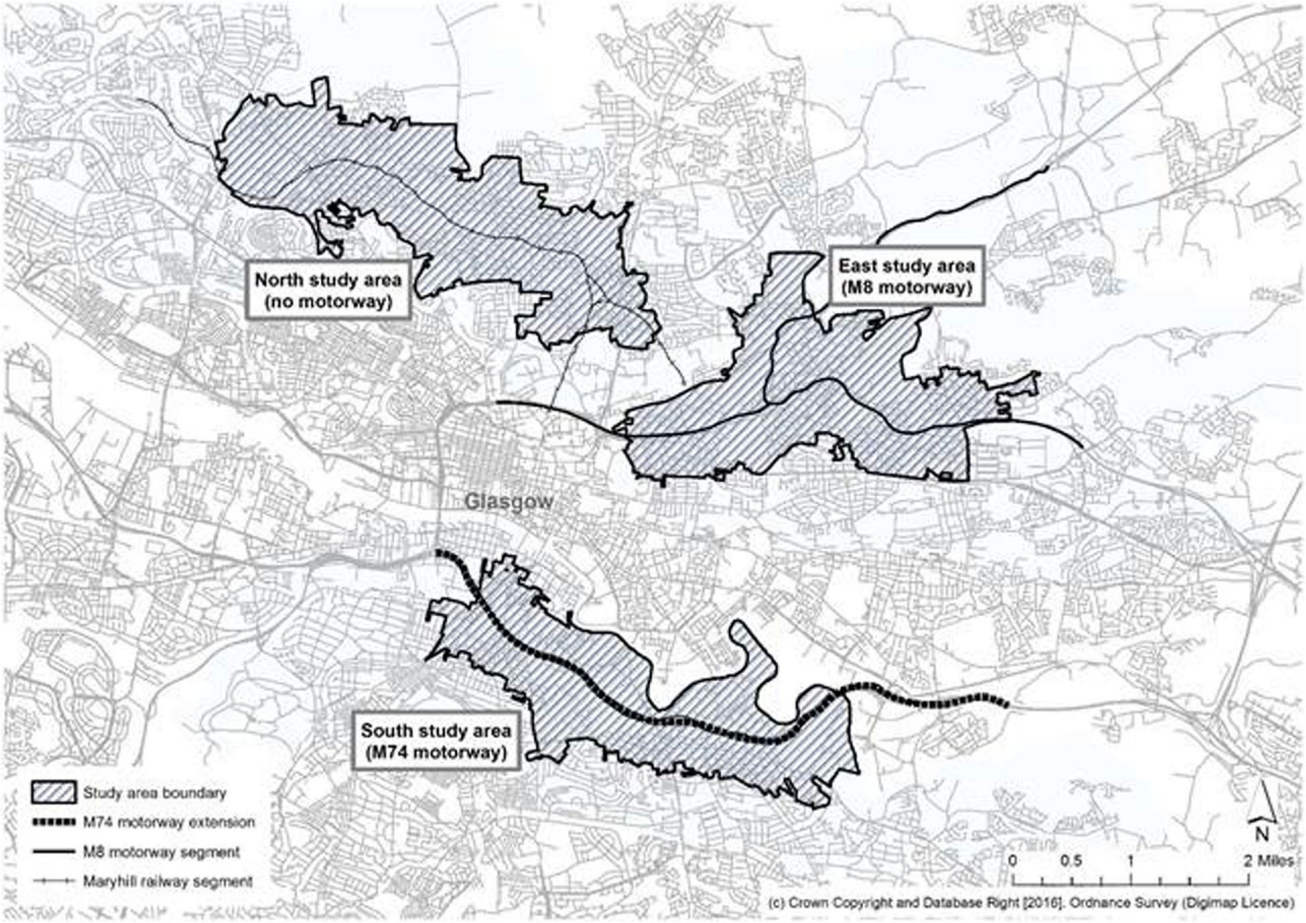
Boundaries of local study areas defined in terms of Census output areas at baseline

The ‘M74 corridor’ study area (South) was compared to two other study areas, one surrounding the existing M8 motorway (East) and a third, control, area with no motorway surrounding a quiet suburban railway (North) (Fig. 1). Prior to baseline data collection, these areas had been iteratively delineated in a Geographic Information System (GIS) using routinely available data and field visits to ensure broadly similar sociodemographic and built environmental characteristics [14]. The study areas were defined as census output areas lying wholly or partly within a 500 m buffer surrounding the M74 (South), the M8 (East) or the suburban railway (North). To ensure that the areas were similar, aggregate census data including levels of unemployment and deprivation, car and home ownership and chronic illness were summarised for the M74 (South) study area. The boundaries of the other two areas were iteratively adjusted in the GIS until they matched the M74 (South) study area on these characteristics.

At baseline there were no statistically significant differences between the areas on socioeconomic or behavioural measures apart from a difference of borderline statistical significance (*p* = 0.053) in the distribution of housing tenure [15]. The three study areas had a similar mix of housing stock, with high-density tenements, high-rise flats and new housing developments [15].

The analyses described in this paper are focussed on secondary outcome measures for the main study, for which no a priori sample size estimations were made. For the primary outcomes (e.g. minutes walked for transport, measured by a travel diary), an a priori sample size estimation was made. For these measures, a total of 400 participants per study area at each time point was expected to allow the detection of an increase of five minutes’ walking for transport per day with 95% confidence and 80% power [16].

#### Sample

The study sample comprised a longitudinal cohort and two distinct cross-sectional samples, recruited from the three study areas at baseline (T1) in 2005 and follow-up (T2) in 2013. At baseline, all residential addresses in each of the three study areas were identified from the Royal Mail Postcode Address File (PAF, version 2005.3). In each study area, 3000 addresses (i.e. 9000 in total) were randomly selected and were mailed a survey in the first week of October 2005 [15] to be completed by a quasi-randomly selected adult member of the household. At follow-up, surveys were sent in the same week of the year to all baseline participants who could still be contacted, including those who had moved between or out of the study areas but not out of the United Kingdom. This sample was topped up to 3000 per area (i.e. 9000 in total) with a new random sample of cross-sectional participants, identified from the updated PAF. Participants who did not live in one of the study areas at follow-up were excluded from the analyses.

To maximise response to the surveys, potential participants were sent a notification postcard a week in advance [17] of the survey and study documentation, and the survey was re-sent to all non-responding households approximately one month later. Respondents were entered into a £50 prize draw (baseline) or received a £5 voucher (follow-up).

The study was approved by University of Glasgow Ethics Committees (see *Ethical approval*). Baseline participants were given the opportunity to complete an optional consent form to allow them to be contacted for follow-up, but the ethical approval allowed for survey data to be used for analysis without such explicit consent on the basis that returning the survey indicated implicit consent.

### Measures

#### Moderate-to-vigorous physical activity, walking and sedentary behaviour

The survey included questions on demographic and socioeconomic characteristics and various health-related outcomes that were hypothesized to be influenced by exposure to a motorway (travel behaviour, physical activity, wellbeing, and perceptions of the local neighbourhood).To minimise participant burden, physical activity and sedentary behaviour were measured with the short International Physical Activity Questionnaire (IPAQ) at baseline and follow-up. Participants reported the number of minutes per day and the number of days per week in which they engaged in moderate intensity activity (MPA), vigorous intensity activity (VPA) and walking. They also reported the time they usually spent sitting on a weekday. The IPAQ has acceptable validity and reliability [18].

IPAQ data were cleaned following standard procedures [19]. Weekly minutes spent in MPA, VPA and walking were calculated by multiplying the average daily time by the weekly frequency, and MPA and VPA were summed to derive the time spent in moderate-to-vigorous physical activity (MVPA). Sedentary behaviour was defined as the time the respondents usually spent sitting on a weekday.

#### Exposure

Exposure in natural experimental studies is a complex concept and is sometimes best captured using measures of differential exposure (e.g. based on individual distance from an environmental change) in combination with dichotomous measures (e.g. living in an “exposed” area with an environmental change or in an “unexposed” area without such a change) [20]. We therefore used two complementary measures of motorway exposure. The first was the study area from which each participant was sampled (i.e. new motorway (South), existing motorway (East) or no motorway (North)). However, individual exposure to a motorway also differs within neighbourhoods and the consequences (e.g. the amount of traffic on local roads) may differ according to distance from the motorway. Therefore, a second measure of exposure was computed for each participant within their study area, using the road network distance in metres from the weighted population centroid of their unit postcode (the smallest unit of postal geography in the UK, covering about 15 residential addresses on average) to the nearest motorway junction (on either the M8 or the M74). It was hypothesized that the effect of a unit change in exposure on the outcomes would be greater among those living closer to motorway infrastructure [21]. Therefore, individual motorway exposure was defined as the negative of the natural logarithm of this distance, so that higher values reflected greater exposure, and was analysed stratified by study area.

#### Covariates

At baseline and follow-up, participants reported their gender, age, housing tenure, number of cars owned, working status and years lived in the neighbourhood. For the purpose of analysis, housing tenure was dichotomized as rental versus ownership, car ownership as not owning a car versus owning at least one car, and working status as not working versus working or studying. Detailed information on the distribution of the covariates is shown in Additional File 1: Table S1.

### Analyses

Analyses were conducted separately on the cohort sample and the repeat-cross sectional sample, because data were available at two time points for each participant in the cohort sample, but only one time point per participant in the repeat-cross sectional sample.

#### Descriptive analysis

Differences in covariates and outcomes between the study areas at baseline and follow-up, and between the cohort and repeat cross-sectional samples, were studied using one-way analysis of variance and chi-square tests as appropriate. For the cohort, potential attrition bias was studied by regressing an indicator of attrition on sociodemographic variables.

#### Two-part regression models of MVPA and walking

For time spent in MVPA and walking we used two-part models [22–24]. These are based on two hypothesized processes. The first is that which determines whether a participant engages in a behaviour (such as walking) or not. This was modelled using a logistic regression. The second, which is conditional on the participant having reported a given behaviour, is that which determines the quantity of that behaviour (such as the time spent walking, among those who walk). The latter data were skewed, and the second process was therefore modelled using a generalized linear model (GLM) with a gamma family and log link. The same covariates were used in each part of the model. For a two-part model it is important that the zero values are genuine and do not simply reflect non-response. Our walking and MVPA summary variables showed a comparatively high number of zero counts, which were not due to non-response but were positively reported as zero by the participants.

#### Linear regression models of sedentary behaviour

Sedentary behaviour was normally distributed, with very few people reporting no sedentary behaviour, and is not readily conceptualised as a two-stage process. It was therefore modelled using linear regression.

#### Modelling differences over time in the cohort sample

To model changes in outcomes in the cohort, the value of each outcome at follow-up was regressed on the baseline value of the outcome, motorway exposure at follow-up was entered as the main independent variable of interest, and the analyses were adjusted for the baseline values of the covariates. In separate models, exposure to the motorway was conceptualised as area-level exposure or as individual exposure stratified by study area. Only participants with full data on all covariates were included in the analyses.

#### Modelling differences over time in the repeat cross-sectional sample

For the repeat cross-sectional analyses, a variable indicating the time point (baseline or follow-up) of each measurement was added. The statistical interaction between time point and motorway exposure at that time point indicated the extent to which exposure was related to a change in the outcome at a population level over time. Each outcome was regressed on this statistical interaction and the covariates that had been measured at the relevant time point. In separate models, exposure to the motorway was conceptualised as area-level exposure or individual exposure stratified by study area. Only participants with full data on all covariates were included in the analyses.

## Results

### Description of the samples

#### Response rate

In total 1345 completed surveys (representing a 16.1% response ratio) were returned at baseline and 1343 (15.8%) at follow-up. The cohort consisted of 365 participants and the other participants (980 at baseline and 978 at follow-up) comprised the repeat cross-sectional sample (Table 1). Older participants (odds ratio (OR) 1.02; 95% confidence interval (CI) 1.01, 1.03) and those working or studying (OR 1.83; 95%CI: 1.30, 2.59) at baseline were more likely to remain in the cohort than younger participants and those not working or studying.

**Table 1:** Socio-demographic and behavioural characteristics of the cohort and repeat cross-sectional samples and differences between time points

	Cohort			Repeat cross-sectional			
	T1 Mean (SD) / %	T2 Mean (SD) / %	N	T1 Mean (SD) / %	N	T2 Mean (SD) / %	N
Age	**50.4 (13.6)**	**58.4 (13.6)**	358	**48.8 (18.3)**	962	**52.6 (16.5)**	970
% Male	43.7%	43.7%	359	**37.1%**	970	**42.8%**	972
% Home ownership	61.2%	63.1%	358	47.9%	965	49.6%	971
% Car ownership	58.4%	60.1%	358	**48.8%**	951	**53.4%**	969
% “Working”	**58.7%**	**48.6%**	358	48.3%	961	48.3%	972
Years lived in local area	18.3 (15.3)	24.9 (16.6)	362	18.2 (18.0)	980	19.0 (17.4)	965
% who walked	88.6%	86.6%	254	81.4%	753	82.0%	793
Walking time if walked (min/week)	376.6 (353.3)	392.9 (366.9)		**410.3 (392.9)**		**355.22 (346.3)**	
% who participated in MVPA	75.8%	76.3%	219	65.4%	694	70.2%	749
MVPA time if participated in MVPA (min/week)	501.4 (500.2)	529.0 (508.9)		569.8 (506.2)		513.9 (447.6)	
Sedentary time (min/day)	402.7 (234.0)	388.1 (229.2)	220	380.6 (247.6)	649	380.7 (239.5)	711

Bold values represent statistically significant differences between time points (*p* < 0.05). *SD* standard deviation, *MVPA* moderate-to-vigorous physical activity, *N* number of observations

#### Differences between study area samples at baseline and follow-up

Previously published analyses of baseline data found no significant differences in socioeconomic or behavioural measures between the samples recruited from the three study areas [15]. At follow-up, participants in the repeat cross-sectional sample living in the North (no motorway) tended to be older (with a mean age of 54.3 years) than those living in the South (new motorway) (51.2) or East (existing motorway) (51.6), and those living in the South (new motorway) tended to have lived in their neighbourhood for less time (a mean duration of 16.3 years) than those in the East (existing motorway) (20.6) or North (no motorway) (19.6).

#### Differences between the two cross-sectional samples

In the repeat cross-sectional sample, weekly time spent walking was lower at follow-up than at baseline (355.1 vs 410.3 min; Table 1). On average, the follow-up sample was older than the baseline sample (52.6 vs 48.8 years) and included a higher proportion of men (42.8% vs 37.1%) and of car owners (53.4% vs 48.8%) (Table 1).

### Main results

#### Cohort analysis

In the cohort sample, the proportion of participants who reported any walking decreased slightly over time in all areas. The proportion who reported any MVPA also decreased in the areas with an existing motorway (East; from 73.8% to 65.6%) or a new motorway (South; from 77.8% to 75.0%), whereas this proportion increased in the control area (North; from 62.0% to 68.5%). On average the daily time spent sitting increased in the South (from 398.5 to 402.0 min), whereas it decreased minimally in the East (from 376.5 to 375.6 min) and more so in the North (from 382.6 to 367.1 min) study areas (Additional file 1: Table S2).

Table 2 shows the main results of the multivariable regression analyses for the cohort. After adjustment for MVPA at baseline, participants living in the study area with an existing motorway (East) were less likely to report participation in MVPA at follow-up than those living in the control (North) area without a motorway (OR 0.37; 95%CI 0.15, 0.91). Within the area with an existing motorway (East), participants living closer to a motorway junction were less likely than those living further away to report participating in MVPA at follow-up (OR 0.30; 95%CI: 0.09, 0.97). Among those who reported any MVPA, no associations between time spent in MVPA and motorway exposure were found. No statistically significant differences in outcomes were found for walking or sedentary behaviour.

**Table 2:** Longitudinal associations between motorway exposure and change in walking, moderate-to-vigorous physical activity and sedentary behaviour

	Walking			MVPA			Sedentary behaviour	
		Participation (yes/no)	Minutes per week		Participation (yes/no)	Minutes per week		Minutes per day
Exposure	N	OR (95% CI)	IRR (95% CI)	N	OR (95% CI)	IRR (95% CI)	N	B (95% CI)
Area: New motorway (South) (Reference: no motorway (North))	248	0.68 (0.24, 1.89)	0.82 (0.62,1.10)	214	0.60 (0.25, 1.43)	0.94 (0.68, 1.31)	215	52.46 (−15.70, 120.62)
Proximity within study area with new motorway	88	1.54 (0.24, 9.70)	1.27 (0.86,1.89)	70	2.39 (0.49, 11.65)	1.27 (0.76, 2.12)	81	−40.17 (−125.38, 45.05)
Area: Existing motorway (East) (Reference: no motorway (North))	248	0.57 (0.19, 1.68)	1.07 (0.79,1.47)	214	**0.37 (0.15, 0.91)**	0.89 (0.62, 1.27)	215	39.39 (−33.48, 112.27)
Proximity within study area with existing motorway	69	1.55 (0.32, 7.52)	1.03 (0.71,1.50)	59	**0.30 (0.09, 0.97)**	0.77 (0.46, 1.29)	59	59.41 (−26.91, 145.73)

Bold values represent statistically significant associations (*p* < 0.05). MVPA = moderate-to-vigorous physical activity, *OR* odds ratio, *IRR* incidence rate ratio, *B* beta, *CI* confidence interval

Proximity was defined as the negative of the natural logarithm of the road network distance in metres from the weighted population centroid of the unit postcode of residence to the nearest motorway junction. Analyses were adjusted for age, gender, home ownership, car ownership, work status and time lived in neighbourhood

#### Repeat cross-sectional analysis

In the repeat cross-sectional sample, the proportion of participants who reported any walking was stable over time in all three study areas. The proportion who engaged in any MVPA increased in the area with a new motorway (South) and the area with no motorway (North), whereas it remained stable in the area with an existing motorway (East). In the area with a new motorway (South), among those who reported any MVPA, the average time spent in MVPA increased slightly over time, whereas it was stable in the area with an existing motorway (East) and decreased in the area with no motorway (North) (Additional file 1: Table S3).

The multivariable regression analyses showed no statistically significant differences in the outcomes in the repeat-cross sectional sample (Table 3). Some of the outcomes were similarly patterned by study area as in the cohort: compared to the area with no motorway (North), in the areas with a new (South) or existing (East) motorway, the time spent sitting increased.

**Table 3:** Repeat cross-sectional associations between motorway exposure and change in walking, moderate-to-vigorous physical activity and sedentary behaviour

	Walking			MVPA			Sedentary behaviour	
		Participation (yes/no)	Minutes per week		Participation (yes/no)	Minutes per week		Minutes per day
Exposure	N	OR (95% CI)	IRR (95% CI)	N	OR (95% CI)	IRR (95% CI)	N	B (95% CI)
Area: New motorway (South) (Reference: no motorway (North))	1499	0.95 (0.47, 1.93)	1.08 (0.83, 1.40)	1412	0.95 (0.53, 1.72)	0.94 (0.71, 1.25)	1318	20.72 (−42.59, 84.03)
Proximity within study area with new motorway	475	0.46 (0.12, 1.70)	0.89 (0.56, 1.42)	450	0.36 (0.12, 1.05)	0.85 (0.53, 1.37)	431	38.65 (−73.48, 150.79)
Area: Existing motorway (East) (Reference: no motorway (North))	1499	1.00 (0.53, 1.92)	1.05 (0.81, 1.38)	1412	0.67 (0.37, 1.22)	1.01 (0.77, 1.32)	1318	16.18 (−47.30, 79.66)
Proximity within study area with existing motorway	495	1.50 (0.59, 3.80)	1.42 (0.95, 2.12)	474	1.05 (0.44, 2.48)	1.36 (0.93, 1.98)	432	35.86 (−52.98, 124.70)

*MVPA* moderate-to-vigorous physical activity, *OR* odds ratio, *IRR* incidence rate ratio, *B* beta, *CI* confidence interval. Proximity was defined as the negative of the natural logarithm of the road network distance in metres from the weighted population centroid of the unit postcode of residence to the nearest motorway junction. Analyses were adjusted for age, gender, home ownership, car ownership, work status and time lived in neighbourhood

## Discussion

### Principal findings

We found some evidence for a reduction in physical activity participation among people living in the study area surrounding the existing M8 motorway, and within this area, greater proximity to the motorway was associated with a reduced likelihood of participating in MVPA over time. We did not find statistically significant changes in MVPA, walking or sedentary behaviour among people exposed to the new M74 motorway extension.

### Strengths and limitations of the study

This is the first study to have evaluated the effects of motorways on physical activity and sedentary behaviour, and among the first to have evaluated the effects of any environmental change on these behaviours using robust quasi-experimental methods. We reported both individual and population level changes in multiple related outcomes, and investigated their associations with multiple measures of exposure using a combination of between-area and within-area analyses. A further strength is the use of two-part models to deal with the non-normal distributions of MVPA and walking. Despite its good theoretical and statistical fit, this technique has not often been applied in physical activity and public health research. A limitation of such models is that despite the good overall statistical fit, the power of the second part of the models is dependent on the number of people with non-zero values for the outcome.

We also acknowledge the more general limitations of this study. First, the representativeness of the study samples should be considered. The study areas included some of the most socioeconomically deprived areas of Glasgow. Although the various indicators of socioeconomic status show that on average the sample had a low socioeconomic position, self-selection bias may still have occurred. Thus, we make no claim that our sample is representative of the source population. Attrition in the cohort was substantial, albeit similar to that of other studies [25]. Those remaining in the cohort were on average older and more likely to work than the rest of the baseline sample. Our analyses were adjusted for a range of potential confounders, and to further offset the limitations of the cohort we recruited an additional sample at follow–up to enable a complementary set of repeat cross-sectional analyses. Second, we used self-reported measures of physical activity and sedentary behaviour (the short IPAQ). The combination of large standard deviations in these measures and the low cohort sample size reduced the statistical power of the study to detect effects, although it is equally plausible that the lack of significant findings on walking and sedentary behaviour may reflect a true absence of association. Although we controlled for socio-economic differences in both the study design (by delineating areas in such a way that the samples were comparable in aggregate socio-economic and behavioural variables) and by adjusting the analyses for indicators of individual socio-economic position (i.e. housing tenure, car ownership and working status), a third limitation is that we omitted some other aspects of individual socio-economic position such as educational attainment. A final limitation is the possibility of unmeasured confounding. We minimised this by carefully delineating the study areas to ensure the comparability of the samples and settings and by adjusting the analyses for multiple sociodemographic covariates. However, it remains possible that other actions may have been taken in some neighbourhoods that directly or indirectly influenced local activity patterns. A complementary programme of qualitative research will explore this further.

### Comparison with previous studies

With no similar previous studies available [11, 26], direct comparisons with the existing literature are not straightforward. However, exposure to road traffic noise has been found to be associated with less time spent outdoors [27], greater physical inactivity [28] and greater prevalence of overweight [29, 30], and a Canadian study has indicated that adults perceive routes away from traffic noise to be more attractive for cycling [31]. On the other hand, a cross-country ecological comparison has shown an inverse relationship between the presence of motorways and the national prevalence of overweight and obesity [32]. That finding may reflect residual confounding from other national characteristics, such as economic prosperity, that may independently influence both the development of the road network and overweight. It is not necessarily in conflict with a finding that among people living close to those motorways, exposure to the infrastructure may be associated with poorer health (including less healthy behaviour patterns) and thereby contribute to social inequalities in health.

The effects of motorway exposure in the cohort sample were stronger in the area with the existing M8 motorway than in the area with the new M74 extension. This finding is consistent with — although not proof of — a temporal dose-response relationship whereby the impacts of the M74 extension may not have not fully developed in the two years that had elapsed between the opening of the motorway and the collection of follow-up data. In previous work, it was hypothesized that the introduction of the M74 might catalyse other changes in the neighbourhoods affected, including wider regeneration. These potential impacts were articulated in two vignettes: one suggesting a virtuous cycle and one a vicious cycle [13]. It is likely that impacts of this kind would take time to emerge. Such an interpretation is congruent with the idea that changes in a system may produce non-linear effects over time. Furthermore, such an interpretation is congruent with the idea that changes in a system may produce non-linear effects over time [33] — as was previously shown in an evaluation of new walking and cycling routes elsewhere in the UK, in which significant effects on physical activity were observed after two years but not after one year [34]. In addition to this evidence for a *temporal* dose-response relationship, the observation that participants in this area who lived closer to the motorway were more likely to discontinue MVPA than those living further away indicates a potential *spatial* dose-response relationship. The context-dependency of the causal processes linking environmental change with behaviour change may explain the inconsistent patterns of associations *within* study areas, because some contextual factors such as the presence of shops or social cohesion may be differently distributed in space; and as these local systems evolve over time, changing temporal and spatial exposures may interact with each other producing different patterns of outcomes. To fully understand the complex temporal and spatial dynamics in such systems other techniques, such as agent-based modelling, might be used. In addition to gaining deeper conceptual insight into the process of change, the results of such studies may also inform the optimal timing of follow-up measurements in future studies like ours.

### Implications for policy and research

Our findings suggest that motorways may have limited impacts on physical activity and sedentary behaviour overall. This may reflect a complex, interwoven mixture of advantages (e.g. more economic activity in the neighbourhood) and disadvantages (e.g. severance from amenities, increased local traffic and more time spent sitting in cars) that may co-evolve over time and largely cancel each other out [13]. Together with other previous research, however, the balance of our findings indicate the potential for new major road infrastructure focused on improving conditions for motor vehicle traffic to have deleterious consequences for health-related behaviours such as physical and sedentary activities. These insights may be particularly important for (mostly lower- and middle-income) countries and regions going through a ‘motorisation transition’ [35] involving the rapid construction or expansion of highway networks. However, the aforementioned possibility of complex and non-linear effects poses a challenge for the generalisation of findings between settings with different contexts. This challenge might be addressed in several complementary ways: by seeking to cumulate evidence from more outcome evaluations conducted in a variety of settings; by investigating the mechanisms by which these environmental changes ‘work’, using a combination of qualitative and quantitative methods [21, 36, 37] and by using simulation modelling to further explore the hypothesized complex and non-linear effects in systems [38].

## Conclusions

We found some evidence of a negative impact of urban motorway infrastructure on physical activity. However, many of the associations we found were weak and not statistically significant. The impact of motorways on physical activity, sedentary behaviour and other health outcomes is likely to be complex and context-dependent and may also be non-linear, both temporally and spatially. To better inform urban transport and planning policy, future studies should investigate the complexity of these effects and mechanisms while acknowledging that physical activity is only one of a set of multiple related outcomes of importance for population health. Further analyses of changes in travel behaviour and wellbeing related to motorway exposure in this study will contribute to understanding this broader set of outcomes.

## Additional file

Additional file 1: Summary statistics for covariates and outcome variables by study area. **Table S1.** Covariates at T1 and T2 by study area for the cohort and repeat-cross sectional samples. **Table S2.** Outcome variables at T1 and T2 by study area for the cohort sample. **Table S3.** Outcome variables at T1 and T2 by study area for the repeat cross-sectional sample. (DOCX 19 kb)
