# Supplementary material for: Effects of urban motorways on physical activity and sedentary behaviour in local residents: a natural experimental study
Source: Int J Behav Nutr Phys Act. 2017 Jul 27;14:102. doi: 10.1186/s12966-017-0557-0 (PMC5530966; doi:10.1186/s12966-017-0557-0)
Supplement: Additional file 1: — Summary statistics for covariates and outcome variables by study area. Table S1. Covariates at T1 and T2 by study area for the cohort and repeat-cross sectional samples. Table S2. Outcome variables at T1 and T2 by study area for the cohort sample. Table S3. Outcome variables at T1 and T2 by study area for the repeat cross-sectional sample. (DOCX 19 kb) [file 12966_2017_557_MOESM1_ESM.docx]

**Additional file 1. Summary statistics for covariates and outcome variables by study area**

**Table S1.** Covariates at T1 and T2 by study area for the cohort and repeat-cross sectional samples

| **Variable** | **Longitudinal cohort**  (n=365) | | | | **Repeat cross-sectional sample**  (T1 n=980; T2 n=978) | | | |
| --- | --- | --- | --- | --- | --- | --- | --- | --- |
|  | T1 | | T2 | | T1 | | T2 | |
|  | *n* | *mean (SD)* | *n* | *mean (SD)* | *n* | *mean (SD)* | *n* | *mean (SD)* |
| Age (years) |  |  |  |  |  |  |  |  |
| Total | 360 | 50.4 (13.6) | 363 | 58.5 (13.6) | 962 | 48.8 (18.3) | 970 | 52.6 (16.5)** |
| New motorway (South) | 125 | 51.0 (14.1) | 125 | 59.0 (14.1) | 312 | 48.1 (17.8) | 304 | 51.2 (16.4) |
| Existing motorway (East) | 111 | 51.3 (13.3) | 112 | 59.4 (13.3) | 317 | 48.5 (18.7) | 329 | 51.8 (17.0) |
| No motorway (North) | 124 | 49.0 (13.3) | 126 | 57.3 (13.4) | 333 | 49.7 (18.2) | 337 | 54.6 (16.0) |
| % male |  |  |  |  |  |  |  |  |
| Total | 361 | 43.5 | 363 | 44.4 | 970 | 37.1 | 972 | 42.8 |
| New motorway (South) | 125 | 48.8 | 125 | 49.6 | 315 | 41.3 | 304 | 45.1 |
| Existing motorway (East) | 111 | 44.1 | 112 | 44.6 | 318 | 34.0 | 331 | 40.2 |
| No motorway (North) | 125 | 37.6 | 126 | 38.9 | 337 | 36.2 | 337 | 43.3 |
| % home ownership |  |  |  |  |  |  |  |  |
| Total | 360 | 61.1 | 363 | 62.5 | 965 | 47.9 | 971 | 49.6 |
| New motorway (South) | 124 | 61.3 | 125 | 62.4 | 315 | 46.4 | 304 | 50.0 |
| Existing motorway (East) | 111 | 61.3 | 112 | 62.5 | 313 | 51.1 | 331 | 48.6 |
| No motorway (North) | 125 | 60.8 | 126 | 62.7 | 337 | 46.3 | 336 | 50.3 |
| % car ownership |  |  |  |  |  |  |  |  |
| Total | 361 | 58.5 | 362 | 60.5 | 951 | 48.8 | 969 | 53.4 |
| New motorway (South) | 125 | 60.8 | 124 | 59.7 | 307 | 47.6 | 304 | 53.0 |
| Existing motorway (East) | 111 | 52.3 | 112 | 55.4 | 312 | 49.4 | 329 | 52.3 |
| No motorway (North) | 125 | 61.6 | 126 | 65.9 | 332 | 49.4 | 336 | 54.8 |
| % working* |  |  |  |  |  |  |  |  |
| Total | 359 | 58.5 | 364 | 48.1 | 961 | 48.3 | 972 | 48.3 |
| New motorway (South) | 124 | 59.7 | 125 | 47.2 | 313 | 48.9 | 304 | 51.0 |
| Existing motorway (East) | 110 | 54.6 | 112 | 46.4 | 315 | 48.9 | 330 | 49.7 |
| No motorway (North) | 125 | 60.8 | 127 | 50.4 | 333 | 47.2 | 338 | 44.4 |
| Years lived in local area |  |  |  |  |  |  |  |  |
| Total | 365 | 18.3 (15.3) | 362 | 24.9 (16.6) | 980 | 18.2 (18.0) | 965 | 19.0 (17.4)** |
| New motorway (South) | 126 | 20.3 (18.4) | 126 | 27.0 (20.3) | 323 | 17.3 (18.4) | 303 | 16.3 (17.1) |
| Existing motorway (East) | 112 | 17.5 (13.5) | 110 | 24.9 (14.0) | 319 | 18.2 (16.9) | 330 | 20.7 (18.1) |
| No motorway (North) | 127 | 16.9 (13.1) | 126 | 22.7 (14.1) | 338 | 18.9 (18.7) | 332 | 19.7 (16.9) |

n – number; T – time point; SD – standard deviation;

*In paid employment (full or part-time), full-time student, or undertaking voluntary work

**Significant difference between study areas within the same time point and study sample (p<0.05)

**Table S2.** Outcome variables at T1 and T2 by study area for the cohort sample

|  | **New motorway (South)** |  |  | **Existing motorway (East)** |  |  | **No motorway (North)** |  |  |
| --- | --- | --- | --- | --- | --- | --- | --- | --- | --- |
|  | **T1**  **Mean (SD) / %** | **T2**  **Mean (SD) / %** | **N** | **T1**  **Mean (SD) / %** | **T2**  **Mean (SD) / %** | **N** | **T1**  **Mean (SD) / %** | **T2**  **Mean (SD) / %** | **N** |
| % who walked | 88.8% | 86.5% | 89 | 87.3% | 84.5% | 71 | 89.8% | 88.6% | 88 |
| Walking time if walked (min/week) | 401.7 (366.9) | 346.3 (328.7) | 72 | 376.1 (347.8) | 447. 9 (393.3) | 57 | 380.6 (361.5) | 384.7 (381.9) | 73 |
| % who participated in MVPA | 77.8% | 75.0% | 72 | 73.8% | 65.6% | 61 | 75.3% | 84.0% | 81 |
| MVPA time if participated in MVPA (min/week) | 568.8 (508.9) | 504.1 (401.8) | 49 | 473.9 (485.8) | 525.7 (458.2) | 35 | 436.5 (466.7) | 563.5 (634.4) | 55 |
| Sedentary time (min/day) | 391.7 (213.9) | 405.3 (247.3) | 82 | 403.5 (260.7) | 397.9 (226.2) | 61 | 428.8 (227.8) | 367.2 (210.9) | 72 |

SD=standard deviation. MVPA = moderate-to-vigorous physical activity

**Table S3.** Outcome variables at T and T2 by study area for the repeat cross-sectional sample

|  | **New motorway (South)** |  |  |  | **Existing motorway (East)** |  |  |  | **No motorway (North)** |  |  |  |
| --- | --- | --- | --- | --- | --- | --- | --- | --- | --- | --- | --- | --- |
|  | **T1 Mean (SD) / %** | **T1 N** | **T2 Mean (SD) / %** | **T2 N** | **T1 Mean (SD) / %** | **T1 N** | **T2 Mean (SD) / %** | **T2 N** | **T1 Mean (SD) / %** | **T1 N** | **T2 Mean (SD) / %** | **T2 N** |
| % who walked | 86.3% | 233 | 85.6% | 243 | 79.4% | 233 | 79.9% | 264 | 81.3% | 262 | 81.7% | 267 |
| Walking time if walked (min/week) | 391.4 (404.7) | 201 | 357.4 (338.0) | 208 | 411.4 (397.8) | 185 | 368.6 (355.3) | 211 | 419.1 (372.6) | 213 | 352.1 (352.2) | 218 |
| % who participated in MVPA | 65.5% | 220 | 71.9% | 231 | 70.7% | 225 | 70.5% | 251 | 62.0% | 234 | 68.5% | 254 |
| MVPA time if participated in MVPA (min/week) | 574.9 (542.8) | 144 | 492.9 (440.1) | 166 | 565.9 (449.3) | 159 | 528.0 (462.1) | 177 | 573.1 (534.2) | 145 | 531.1 (448.7) | 174 |
| Sedentary time (min/day) | 398.5 (253.0) | 203 | 402.1 (247.2) | 228 | 376.5 (235.7) | 192 | 375.6 (236.7) | 241 | 382.6 (245.2) | 227 | 367.1 (226.9) | 228 |

SD=standard deviation. MVPA = moderate-to-vigorous physical activity
